# Oral and anal detection of *Treponema pallidum* DNA among transgender women and travestis with high-titre syphilis in Brazil: findings from the MyTTrA study

**DOI:** 10.1016/j.lana.2026.101691

**Published:** 2026-11-05

**Authors:** Romain Salle, Daniel Jason McCartney, Philippe Alain Grange, Paul Mee, Silvia Costa, Laio Magno, Béatrice Berçot, Maria Amélia de Sousa Mascena Veras, Cécile Bébéar, Pamela Gaspar, Philippe Mayaud, Nicolas Dupin, MyTTrA Study group, Nicolas Dupin, Philippe Alain Grange, Guillaume Ollagnier, Romain Salle, Béatrice Berçot, Aymeric Braille, François Caméléna, Manel Merimèche, Cécile Bébéar, Carla Balcon, Jennifer Guiraud, Arabella Touati, Carla Balcon, Jennifer Guiraud, Arabella Touati, Philippe Mayaud, Gwenda Hughes, Daniel Jason McCartney, Paul Mee, Maria Amélia de Sousa Mascena Veras, Katia Cristina Bassichetto, Gustavo Santa Rosa Saggese, Carla Gianna Luppi, Silvia Figueiredo Costa, Laio Magno, Maria Inês Costa Dourado, Pamela Cristina Gaspar

**Affiliations:** a Centre for Genital and Sexually Transmitted Diseases - Dermatology Department, National Center for Bacterial Sexually Transmitted Infections, Hospital Cochin, APHP, Paris, France; b Cutaneous Biology Team, INSERM U1016-CNRS UMR8104, Cochin Institute, University of Paris Cité, Paris, France; c London School of Hygiene & Tropical Medicine, Faculty of Infectious & Tropical Diseases, London, UK; d Lincoln Institute of Rural and Coastal Health, University of Lincoln, UK; e Departamento de Infectologia e Medicina Tropical, Hospital das Clínicas da Faculdade de Medicina da Universidade de São Paulo, São Paulo, Brazil; f Centres for Antimicrobial Optimisation Network (CAMO-net Brazil), Brazil; g Instituto Gonçalo Moniz, Fundação Oswaldo Cruz, Brazil; h Departamento de Ciências da Vida, Universidade do Estado da Bahia, Salvador, Brazil; i Bacteriology Department, Univ Paris Cité INSERM1137, IAME, National Reference Center for Bacterial Sexually Transmitted Infections, Saint Louis hospital, APHP, Paris, France; j Faculdade de Ciências Médicas da Santa Casa de São Paulo, São Paulo, Brazil; k Department of Bacteriology, National Center for Bacterial Sexually Transmitted Infections, CHU Bordeaux, UMR CNRS 5234 MFP, Univ. Bordeaux, Bordeaux, France; l Department of HIV, AIDS, Tuberculosis, Viral Hepatitis, and Sexually Transmitted Infections, Brazilian Ministry of Health, Brasília, Brazil

**Keywords:** *Treponema pallidum*, Syphilis, Nucleic acid amplification techniques, Oral, Anal, Transgender women, Azithromycin, Antimicrobial resistance

## Abstract

**Background:**

Data on transgender women and *travestis* (TGW) concerning oral and anal detection of *Treponema pallidum* (TP) DNA are lacking. We aimed to estimate the prevalence of oral and anal TP DNA detection and the frequency of azithromycin resistance-associated mutations among TGW with high-titre syphilis in Brazil.

**Methods:**

We conducted a nested cross-sectional study as part of the TransOdara study among TGW in five Brazilian cities. Participants with high-titre syphilis (reactive treponemal test and VDRL titre ≥1:8) and an analysable oral or anal specimen underwent quantitative PCR targeting the *tpp47* and *polA* genes to detect TP DNA. TP DNA-positive specimens were further analysed using nested PCR and Sanger sequencing to identify mutations associated with azithromycin resistance.

**Findings:**

Among the 249 participants with high-titre serological syphilis and analysable sample, TP DNA was detected in 25 (10.0%, 95% CI 6.6–14.5): 14 (5.6%) from the oral site only, 6 (2.4%) from the anal site only, and 5 (2.0%) from both sites. Participants in whom TP DNA was detected were younger and more frequently reported STI symptoms and anogenital ulcers during the preceding six months. The detection rate increased with VDRL titre (odds ratio 2.44, 95% CI 1.74–3.41 per doubling of titre for any oral or anal detection). Mutations associated with azithromycin resistance were observed in 76% (19/25, 95% CI 54.9–90.6) of participants in whom TP was detected.

**Interpretation:**

TP DNA was detected in 10% of participants with high-titre serological syphilis. Detection increased with higher VDRL titres, supporting an association between higher non-treponemal titres and mucosal TP DNA detection. These findings support continued epidemiological and molecular surveillance of syphilis and of azithromycin resistance in transgender populations. They do not support site-specific PCR screening: syphilis diagnosis remains serological, and benzathine benzylpenicillin G remains the treatment of choice.

**Funding:**

French National Agency for Research on AIDS, Viral Hepatitis, and Emerging Infectious Diseases (ANRS/MIE).

**Research in context**

**Evidence before this study**

We searched PubMed and Embase for studies on the molecular detection of *Treponema pallidum* (TP) at mucosal sites published up to October 11, 2026. The following search terms were used: ‘*T. pallidum*’, 'syphilis', ‘PCR’, ‘molecular biology’, ‘oral’, ‘anal’, ‘shedding’, ‘detection’, ‘azithromycin resistance’, ‘transgender’, ‘travesti’, ‘Brazil’ and ‘Latin America’. We also reviewed the reference lists of the retrieved articles. Almost all of the available evidence originates from cisgender men who have sex with men and who were recruited from sexual health services or clinical trials in Australia, United States and Europe. In these studies, the TP DNA detection rate was approximately 25% for early syphilis and around 10% for early latent infection. Lesion, blood and urine specimens, as well as mucosal swabs, were tested. Azithromycin resistance-associated mutations exceed 70% in Europe, North America and China, and have been reported in Brazil. However, Latin American estimates are based on a small number of sequences. One study in Peru has reported the detection of TP DNA in oral and anal swabs from people with clinically diagnosed syphilis, at rates of 8.5% and 38.8%, respectively. To our knowledge, no estimate specific to transgender women or travestis (TGW) has been published.

**Added value of this study**

This study provides molecular estimates of the detection of *T. pallidum* DNA in the oral and anal regions of TGW, from a community-recruited population in Latin America. Among the 249 participants with high-titre syphilis who were recruited from the community across five Brazilian capital cities, TP DNA was detected in 10.0% of cases, with oral involvement observed in 76.0% of those with detectable DNA. TP DNA detection increased with non-treponemal titre, with an odds ratio of 2.44 per doubling of VDRL titre. Azithromycin resistance-associated mutations were present in 76.0% of participants with detectable TP DNA. As participants were identified through serological screening rather than clinical presentation, these estimates reflect a population in which early syphilis presenting clinically was uncommon.

**Implications of all the available evidence**

Mucosal TP DNA is detectable in a measurable minority of TGW identified through community-based serological screening. It is most often found at the oral site and is usually present in the absence of reported symptoms. These data support the continued epidemiological and molecular surveillance of azithromycin resistance in TGW in Latin America, and reinforce the importance of accessible serological screening followed by prompt treatment. However, they do not support site-specific PCR screening, as diagnosis remains serological and PCR detection does not establish viability or infectiousness.

## Introduction

Rates of syphilis, caused by *Treponema* *pallidum* spp. *pallidum* (TP), are increasing worldwide despite the availability of effective screening, diagnostic and therapeutic tools.^1^ Many people with syphilis are asymptomatic or have symptoms that go unrecognised, which may contribute to delayed diagnosis and ongoing undiagnosed infection. Transgender women and *travestis* (TGW) bear a disproportionate burden of sexually transmitted infections, yet they are rarely included in syphilis research beyond serological prevalence surveys. In Brazil, ‘travesti’ denotes a distinct gender identity that is rooted in Latin American social and cultural contexts, rather than being a translation of ‘transgender woman’. Both identities are affected by overlapping forms of social marginalisation. The TransOdara study recruited TGW in five Brazilian capital cities. Of those tested, 22.8% had a reactive treponemal test with a non-treponemal Venereal Disease Research Laboratory (VDRL) titre of 1:8 or higher, most of whom self-identified as Black or mixed race.^2^

Mucosal TP DNA detection, sometimes described as shedding, has been reported at oral and anorectal sites in people with early syphilis, although detection varies by disease stage and study population.^3^, ^4^ These data are derived almost exclusively from cohorts of cisgender men who have sex with men (MSM), recruited from sexual health clinics in Australia, the United States and Europe.^3^, ^4^, ^5^, ^6^ A study in Peru has reported the detection of TP DNA at oral and anal sites in people with a clinical diagnosis of syphilis,^7^ but no specific estimate for TGW has been published.

Moreover, the anatomical site is of particular relevance in this population. In the same TransOdara cohort, infections with *Chlamydia trachomatis* (CT) and *Neisseria gonorrhoeae* (NG) were predominantly extragenital. The prevalence of CT reached 8.5% at the anus and 3.2% at the oropharynx, and the prevalence of NG reached 8.7% and 7.9%, respectively. This compares with prevalence of 0.7% and 0.2%, respectively, in urine specimens.^8^ Most of these infections would have been missed by sampling the genitalia or urine alone. It has not been examined whether TP DNA detection shows a comparable site distribution in TGW.

We therefore examined stored oral and anal specimens from TransOdara participants with high-titre serological syphilis in order to estimate the prevalence and characteristics of TP DNA detection at these anatomical sites, and determine the frequency of azithromycin resistance-associated mutations.

## Methods

### Study design and participants

TransOdara was a cross-sectional study conducted between December 2019 and July 2021 among TGW in five major Brazilian cities: Campo Grande, Manaus, Porto Alegre, Salvador, and São Paulo.^2^ Participants were recruited using respondent-driven sampling (RDS). Initial community “seeds” recruited eligible peers using coded study coupons, with subsequent participants able to recruit further peers. Eligible participants were aged ≥18 years, had been assigned male sex at birth, self-identified with a feminine gender identity, lived, worked, or studied in a participating city, had a referral coupon, and had not previously participated in TransOdara. Although recruitment was conducted across five geographically diverse Brazilian cities, the present analysis was not designed to provide nationally representative estimates for all TGW in Brazil.

### Data and specimen collection

All participants completed a structured interviewer-administered questionnaire addressing sociodemographic characteristics, sexual behaviour, HIV and other sexually transmitted infection (STI) history, prior testing and treatment, as well as current and recent symptoms. Sexual behaviour questions included oral sex and receptive and insertive anal intercourse with each participant's most recent regular, casual and commercial partner in the preceding six months, where applicable. Race and ethnicity were self-reported by participants using the five categories of the Brazilian Institute of Geography and Statistics. Because these questions referred to the last partner in each category, they did not establish the timing of individual exposures relative to syphilis acquisition and were not used in the present analysis. Collection of biological specimens and participation in clinical examination were voluntary, and individual questionnaire items could be left unanswered. Consequently, questionnaire, examination, and specimen data were not complete for all participants. Participants who consented were examined by an infectious diseases' physician, including inspection of the oral cavity and, where separately consented, the genital and anal regions. Anoscopy was not performed and lesions were not swabbed directly for TP DNA; any lesion identified was referred for further evaluation and taken into account in treatment decisions.

Rapid blood tests for HIV, syphilis, hepatitis A, B, and C were offered. Real-time PCR testing for CT and NG was performed on oral and anal swabs and urine using the Abbott RealTime CT/NG assay on the Abbott m2000 platform. Participants could choose self- or provider-collection for oral and anal swabs according to their preference; collection method was not randomised. Participants choosing self-collection were provided with illustrated, site-specific self-collection instructions, and trained study staff were available to clarify the procedure when required.^9^ All specimens used in the present analysis were collected before administration of any STI treatment provided as part of the TransOdara study visit.

For participants with a neovagina following gender-affirming vaginoplasty, neovaginal specimens were used for HPV testing and were not part of CT/NG testing. The present TP molecular analysis was restricted to stored oral and anal swab specimens and did not include urogenital or neovaginal specimens.

### Specimen storage and transfer

Following the original CT/NG testing, residual oral and anal specimens were retained in Abbott Multi-Collect transport medium and stored at −20 °C at the CRT DST/AIDS Laboratory in São Paulo for four to five years until shipment to France in April 2025. Aliquots were shipped frozen under temperature-controlled conditions. Following arrival in France, specimens were stored for approximately four months before DNA extraction, and TP DNA testing was performed 1–2 months later.

### Syphilis serology and case definition

Syphilis testing comprised a rapid treponemal point-of-care test (POCT; Syphilis Assure Test, Assure Tech [Hangzhou] Co., Hangzhou, China, distributed in Brazil by Orbitae Diagnósticos, Brazil) and a quantitative non-treponemal Venereal Disease Research Laboratory (VDRL) test (stabilised antigen suspension, Wiener Laboratorios S.A.I.C., Rosario, Argentina). This last assay is a modified VDRL (unheated serum reagin, USR) test; titres obtained by this method are equivalent to those of the standard VDRL and are reported as VDRL titres throughout. In cases of discordant results (negative POCT and reactive VDRL), a confirmatory fluorescent treponemal antibody absorption test (FTA-Abs; Imuno-CON FTA-Abs Syphilis, Wama Diagnóstica, São Carlos, Brazil) was performed. Serological testing was performed at the Adolfo Lutz Institute, the state reference public health laboratory for São Paulo. Participants with a reactive treponemal test and a VDRL titre of 1:8 or higher were classified as having high-titre syphilis. This threshold is a convention used in epidemiological studies to identify probable active infection where serial titres are unavailable, rather than a diagnostic standard.^10^ It has been applied to asymptomatic participants in a recent molecular study in Mexico,^11^ and it is the definition previously used in the TransOdara cohort.^2^ It also lies immediately above the range in which non-treponemal titres typically stabilise after treatment.^12^ This definition was serological and was not intended to assign a clinical stage of syphilis, which was not determined in this study.

### TP DNA detection and azithromycin resistance-associated mutations

Retrospectively, all available stored oral and anal swab samples from included participants were tested for TP using quantitative PCR (qPCR) targeting the *tpp47* and *polA* genes, as previously described.^13^ TP qPCR was undertaken for epidemiological assessment of mucosal TP DNA detection and molecular surveillance and was not used to establish the diagnosis of syphilis, which was based on serological testing. TP DNA-positive samples were further analysed using nested PCR and Sanger sequencing to identify mutations A2058G or A2059G in the 23S rRNA gene associated with azithromycin resistance following previously established methods.^14^ Mutation status was determined among specimens yielding an interpretable 23S rRNA sequencing result.

### Data linkage and outcome definitions

For the MyTTrA analysis, direct personal identifiers from TransOdara were removed from the analytic dataset. A unique study identifier was retained to permit participant-level linkage of MyTTrA molecular results with corresponding TransOdara questionnaire, clinical, serological, HIV, and other STI data. Analyses were therefore conducted using pseudonymised linked data without access to direct personal identifiers.

Participant-level TP DNA detection was defined as PCR detection at either anatomical site. Site-specific detection was described separately for oral and anal swabs. Participant-level detection patterns were classified according to the available valid site-specific results as oral detection only, anal detection only, detection at both sites, or no TP DNA detected. Participant selection from TransOdara enrolment to the final TP DNA analytic population is summarised in Fig. 1.

**Fig. 1:**
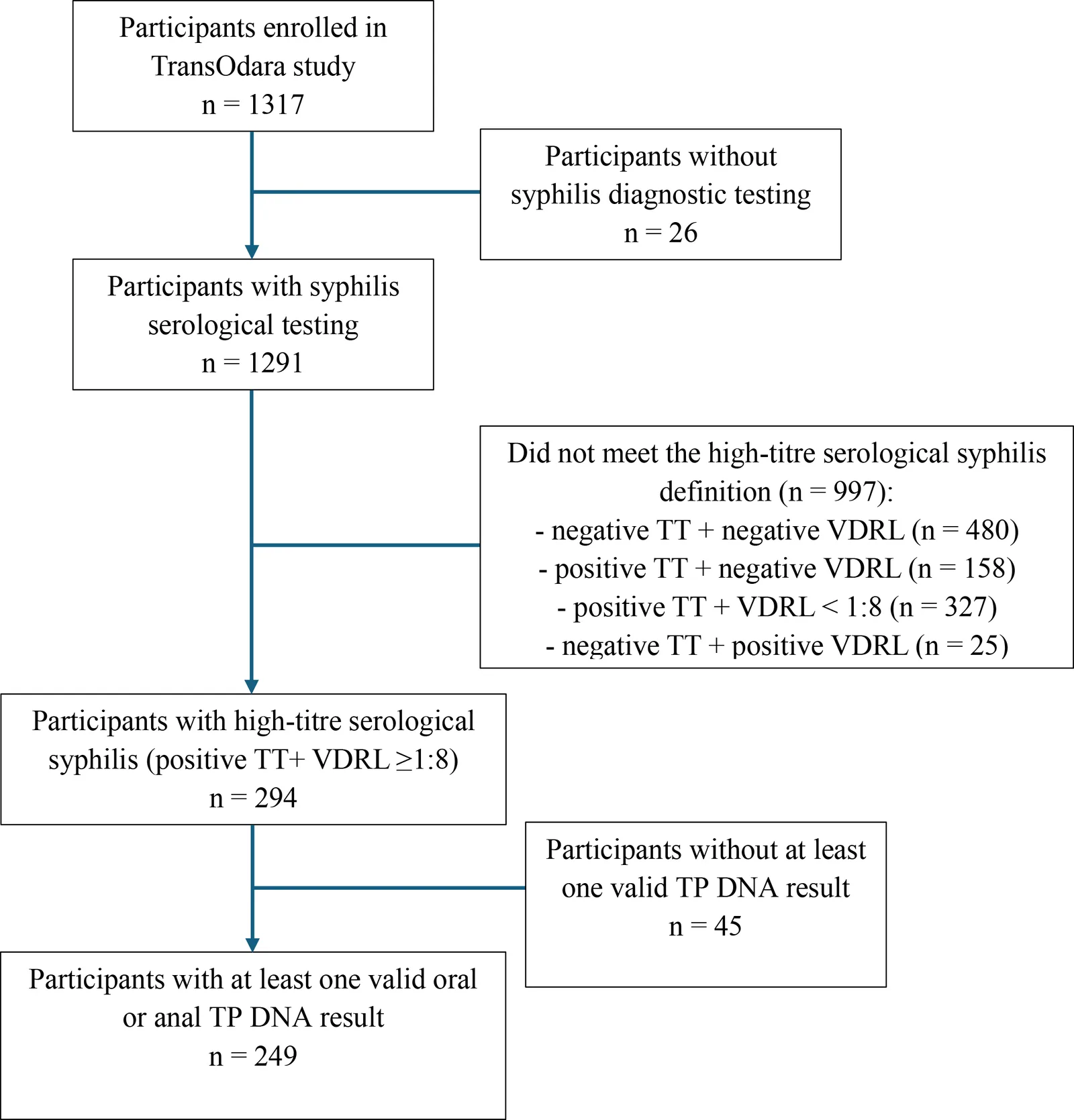
**Flow chart of the study**. Of the 1317 participants enrolled in the TransOdara study, 26 did not undergo syphilis diagnostic testing and 997 did not meet the study's definition of high-titre serological syphilis: a reactive treponemal test with a VDRL titre of 1:8 or higher. Of the 294 participants who met this definition, 45 had no valid oral or anal *Treponema pallidum* DNA test result, leaving a final analysis group of 249 participants. TT, treponemal test; VDRL, Venereal Disease Research Laboratory.

### Ethics

The MyTTrA protocol was approved by the Centro de Referência e Treinamento DST/AIDS, São Paulo, through Plataforma Brasil (CAAE 63984222.5.0000.5375, 23 November 2022), by the Brazilian national Commission for Research Ethics (CONEP, 31 January 2023), and by the LSHTM Research Ethics Committee (no. 30863, 11 June 2024). The parent TransOdara protocol was approved by the Research Ethics Committee of Santa Casa de Misericórdia de São Paulo (CAAE 05585518.7.0000.5479, approval no. 3,126,815, 30 January 2019); as an observational study rather than an interventional trial, TransOdara was not eligible for ReBEC registration. All TransOdara participants consented to specimen collection, long-term storage and future use for purposes connected to the original study aims, and specimens from participants who declined long-term storage were destroyed after TransOdara testing and were not used in MyTTrA. Participants were re-approached for separate MyTTrA consent several years after enrolment, as recommended by CONEP, and consent was obtained from 20% of those who could be traced; a waiver covering pseudonymised data and specimens from all originally consenting participants was therefore granted by CONEP and accepted by the LSHTM Research Ethics Committee. Documentation of the study design, laboratory procedures and standard operating procedures is available at https://nudhes.org/transodara.

### Statistical analysis

Analyses were descriptive and exploratory. Analyses of the selected MyTTrA subset were unweighted, and RDS sampling weights were not applied. The analysis population comprised participants with high-titre syphilis who had at least one valid TP DNA result from an oral or anal swab. Participants without an analysable result at one anatomical site could contribute to analyses at the other site if that result was valid. No imputation was performed for missing questionnaire responses, unavailable specimens, or non-analysable molecular results. Denominators are therefore reported explicitly for each analysis. Unavailable, non-amplified, non-interpretable, and other non-valid specimen results were excluded only from the corresponding site-specific denominator.

Categorical participant characteristics were summarised as frequencies and percentages. Key TP DNA detection and azithromycin resistance-associated mutation proportions were reported with 95% confidence intervals calculated using the Clopper-Pearson exact method, which was preferred because several proportions were equal to or close to 0 or 1 and some denominators were small. Age was summarised as the median and interquartile range and also as the mean and standard deviation.

Characteristics of participants with and without detectable TP DNA were compared using Fisher's exact test for all categorical variables, in preference to Pearson's chi-squared test because expected cell counts fell below five in several comparisons. Odds ratios (OR) for TP detection with exact 95% confidence intervals (CI) were estimated by conditional maximum likelihood. Age was compared using the Wilcoxon rank-sum test, with the Hodges-Lehmann estimate of the median difference and its 95% confidence interval as the effect measure; Welch's *t* test and the corresponding mean difference with 95% CI were reported alongside as a sensitivity analysis.

Trends in TP DNA detection across ordered VDRL titre categories were assessed using the Cochran–Armitage test for trend in proportions, with categories scored by titre rank; because VDRL titres form a doubling series these scores are equivalent to log2 titre values. As the highest titre stratum contained only five participants, a sensitivity analysis repeated the trend tests after excluding this stratum. ORs for detection per doubling of VDRL titre, with 95% CIs, were estimated by logistic regression using the same log2 titre scores.

Participant-characteristic analyses used the available data for the variables required for each comparison; participants were not excluded from other analyses because of missing data on unrelated variables. Missing participant characteristic data were uncommon, affecting no more than 1.2% of participants for any characteristic other than a history of syphilis diagnosis (5.2%). The number of participants with available data is therefore reflected in the denominator for each characteristic. All tests were two-sided. No adjustment was made for multiple comparisons, and results were interpreted primarily according to effect size and precision rather than statistical significance alone. All statistical analyses were conducted using R Statistical Software version 4.5.1.^15^

### Role of the funding source

The funders of the study were not involved in the design of the study, the collection, analysis or interpretation of the data, or the writing of the report.

## Results

A total of 1317 participants were recruited to TransOdara, of whom 1291 underwent syphilis diagnostic testing and 294 (22.8% of those tested) met the study definition of high-titre syphilis. Of these, 249 had at least one valid oral or anal specimen TP DNA result and comprised the TP DNA analysis population, contributing 480 valid site-specific results (242 oral and 238 anal; Fig. 1). Self-reported race or ethnicity was available for 246 of the 249 participants (98.8%). Of these, 116 (47.2%) identified as mixed race (parda), 72 (29.3%) as Black (preta), 53 (21.5%) as White (branca), three (1.2%) as Indigenous (indígena), and two (0.8%) as Asian (amarela). TP DNA was detected in 25/249 participants (10.0%, 95% CI 6.6–14.5), including 14/249 with oral-only detection (5.6%, 95% CI 3.1–9.3), 6/249 with anal-only detection (2.4%, 95% CI 0.9–5.2) and 5/249 with detection at both sites (2.0%, 95% CI 0.7–4.6). Among participants with TP DNA detected, 19/25 (76.0%, 95% CI 54.9–90.6) had oral detection, either alone or together with anal detection (Table 1). Among specimens with a valid TP DNA result and classifiable collection method, oral TP DNA detection was 7.9% (10/126) in self-collected and 7.8% (9/115) in provider-collected specimens (Fisher's exact p = 1.000). Anal detection was 5.4% (10/185) in self-collected and 1.9% (1/52) in provider-collected specimens (p = 0.464). At each site, collection method could not be reliably classified for one specimen with a valid TP DNA result, and these specimens were excluded from this comparison. Among the 30 TP DNA-positive specimens, mean cycle threshold (Ct) values for *polA* and *tpp47* were 29.7 and 26.7 overall, 30.4 and 27.4 in oral specimens, and 28.6 and 25.7 in anal specimens, respectively.

**Table 1:** *Treponema pallidum* detection pattern and azithromycin resistance-associated mutations by VDRL titres, with 95% confidence intervals, among 249 transgender women and travestis with high-titre serological syphilis in Brazil.

	Detection site			
	Oral only n/N % (95% CI)	Anal only n/N % (95% CI)	Oral and anal n/N % (95% CI)	Any oral or anal n/N % (95% CI)
TP and AMR detection				
TP detected	14/249 5.6 (3.1–9.3)	6/249 2.4 (0.9–5.2)	5/249 2.0 (0.7–4.6)	25/249 10.0 (6.6–14.5)
Azithromycin resistance-associated mutations^a^	11/14 78.6 (49.2–95.3)	6/6 100.0 (54.1–100.0)	2/5 40.0 (5.3–85.3)	19/25 76.0 (54.9–90.6)
VDRL titres^b^				
Titre 1:8 (N = 89)	1/89 1.1 (0.0–6.1)	0/89 0.0 (0.0–4.1)	0/89 0.0 (0.0–4.1)	1/89 1.1 (0.0–6.1)
Titre 1:16 (N = 68)	2/68 2.9 (0.4–10.2)	1/68 1.5 (0.0–7.9)	1/68 1.5 (0.0–7.9)	4/68 5.9 (1.6–14.4)
Titre 1:32 (N = 49)	3/49 6.1 (1.3–16.9)	2/49 4.1 (0.5–14.0)	1/49 2.0 (0.1–10.9)	6/49 12.2 (4.6–24.8)
Titre 1:64 (N = 26)	3/26 11.5 (2.4–30.2)	2/26 7.7 (0.9–25.1)	1/26 3.8 (0.1–19.6)	6/26 23.1 (9.0–43.6)
Titre 1:128 (N = 12)	2/12 16.7 (2.1–48.4)	1/12 8.3 (0.2–38.5)	2/12 16.7 (2.1–48.4)	5/12 41.7 (15.2–72.3)
Titre 1:256 (N = 5)	3/5 60.0 (14.7–94.7)	0/5 0.0 (0.0–52.2)	0/5 0.0 (0.0–52.2)	3/5 60.0 (14.7–94.7)
**Test for trend across VDRL titres (Cochran-Armitage p-value)**	<0.001	0.018	0.007	<0.001
**Odds ratio for detection per doubling of VDRL titre (95% CI)**	2.32 (1.56–3.47)	1.83 (1.07–3.15)	2.09 (1.15–3.81)	2.44 (1.74–3.41)

Tests for trend used the Cochran–Armitage test for trend in proportions, with titre strata scored by rank; because VDRL titres form a doubling series these scores are equivalent to log2 titre values. Odds ratios express the change in the odds of TP detection associated with each successive doubling of titre, for example from 1:8 to 1:16, and were estimated by logistic regression using the same scoring. A sensitivity analysis excluding the 1:256 stratum, which contained only five participants, is reported in the Results.

Confidence intervals were calculated using the Clopper-Pearson exact method, which was preferred to normal-approximation (Wald) intervals because several cells contain proportions of 0 or 1, for which Wald intervals have zero width.

AMR: antimicrobial resistance; CI: confidence interval; TP: *Treponema pallidum*; VDRL: Venereal Disease Research Laboratory.

a Azithromycin resistance was defined at participant level as detection of A2058G or A2059G among participants with detected TP DNA and interpretable 23S rRNA sequencing results.

b Row percentages. Denominators for the VDRL titre rows are the number of participants in each titre stratum.

Twenty-two participants (8.8%) reported a current genital or anal sore, or an extragenital lesion, including 5/25 participants (20.0%) with detectable TP DNA. Current anal complaints assessed by the questionnaire included anal discharge, wart, and sore. Oral-specific symptoms were not separately elicited by the questionnaire. Participants with detectable TP DNA were younger than those without detection with estimated age difference of −5.0 years (95% CI −8.0 to −1.0; p = 0.006); the corresponding mean difference was −4.7 years (95% CI −8.5 to −1.0; p = 0.016) (Table 2). Anogenital ulceration in the previous six months was reported by 28.0% of participants with detectable TP DNA versus 4.9% without detection (OR 7.42, 95% CI 2.16–24.18; p < 0.001), and corresponding proportions for any STI symptoms in the previous six months were 44.0% and 21.0% (OR 2.94, 95% CI 1.13–7.51; p = 0.022). Point estimates for a complaint of genital lesion (OR 9.43, 95% CI 0.66–135.79), extragenital lesion (OR 4.15, 95% CI 0.65–19.86) and any symptom suggestive of syphilis (OR 3.02, 95% CI 0.79–9.80) at the time of sampling were also higher in those with detectable TP DNA. Conversely, participants with detectable TP DNA were less likely to report ever having been diagnosed with syphilis (50.0% versus 70.6%; OR 0.42, 95% CI 0.16–1.13), although this characteristic had the greatest proportion of missing data and is interpreted cautiously below. For the remaining characteristics, including HIV serostatus and detection of other bacterial STIs, the confidence intervals were wide and compatible with both no association and moderate associations in either direction; these comparisons are therefore inconclusive rather than negative (Table 2). Clinical examination data were incomplete: genital/anal examination was undertaken in 96/249 (38.6%) participants and oral examination in 150/249 (60.2%).

**Table 2:** Characteristics associated with *Treponema pallidum* DNA detection among 249 transgender women and travestis with high-titre serological syphilis in Brazil.

Variable	Any oral or anal TP detection			Missing n (%)	Effect estimate (95% CI)	p-value
	Total (N = 249) n/N (%)	Positive (N = 25) n/N (%)	Negative (N = 224) n/N (%)			
Age (median, IQR), years	31 (26–37)	26 (22–32)	31 (26–38)	0 (0.0%)	−5.0 (−8.0 to −1.0)^c^	0.006
Age (mean, SD), years	32.3 (9.3)	28.1 (8.7)	32.8 (9.3)	0 (0.0%)	−4.7 (−8.5 to −1.0)^d^	0.016
Behavioural and STI history						
Any sex work partner in past 6 months	126/247 (51.0%)	16/24 (66.7%)	110/223 (49.3%)	2 (0.8%)	2.05 (0.79–5.77)	0.133
Any regular sex partner in past 6 months	120/248 (48.4%)	12/25 (48.0%)	108/223 (48.4%)	1 (0.4%)	0.98 (0.39–2.45)	1.000
Any casual sex partner in past 6 months	101/246 (41.1%)	13/25 (52.0%)	88/221 (39.8%)	3 (1.2%)	1.63 (0.65–4.12)	0.285
Any condomless intercourse in last 72 h	73/248 (29.4%)	6/25 (24.0%)	67/223 (30.0%)	1 (0.4%)	0.74 (0.23–2.03)	0.647
Ever diagnosed with syphilis	162/236 (68.6%)	11/22 (50.0%)	151/214 (70.6%)	13 (5.2%)	0.42 (0.16–1.13)	0.056
Any STI diagnosis in past 6 months	77/249 (30.9%)	6/25 (24.0%)	71/224 (31.7%)	0 (0.0%)	0.68 (0.21–1.87)	0.501
Any syphilis diagnosis in past 6 months	72/249 (28.9%)	5/25 (20.0%)	67/224 (29.9%)	0 (0.0%)	0.59 (0.17–1.70)	0.359
Any STI symptoms in past 6 months	58/249 (23.3%)	11/25 (44.0%)	47/224 (21.0%)	0 (0.0%)	2.94 (1.13–7.51)	0.022
Any anogenital ulcer in past 6 months	18/249 (7.2%)	7/25 (28.0%)	11/224 (4.9%)	0 (0.0%)	7.42 (2.16–24.18)	<0.001
Any genital blister in past 6 months	11/249 (4.4%)	3/25 (12.0%)	8/224 (3.6%)	0 (0.0%)	3.65 (0.58–16.70)	0.086
Current STI symptoms and signs						
Any STI symptoms	43/249 (17.3%)	7/25 (28.0%)	36/224 (16.1%)	0 (0.0%)	2.02 (0.66–5.55)	0.161
Any complaint of genital lesion	4/248 (1.6%)	2/25 (8.0%)	2/223 (0.9%)	1 (0.4%)	9.43 (0.66–135.79)	0.052
Any complaint of anal lesion	9/247 (3.6%)	2/25 (8.0%)	7/222 (3.2%)	2 (0.8%)	2.66 (0.25–15.11)	0.228
Any complaint of extragenital lesion	10/247 (4.0%)	3/25 (12.0%)	7/222 (3.2%)	2 (0.8%)	4.15 (0.65–19.86)	0.068
Any syphilis relevant symptom^a^	22/249 (8.8%)	5/25 (20.0%)	17/224 (7.6%)	0 (0.0%)	3.02 (0.79–9.80)	0.054
Other STIs						
HIV seropositive	116/246 (47.2%)	10/25 (40.0%)	106/221 (48.0%)	3 (1.2%)	0.72 (0.28–1.81)	0.529
Any Neisseria gonorrhoeae (NG)^b^	47/249 (18.9%)	6/25 (24.0%)	41/224 (18.3%)	0 (0.0%)	1.41 (0.43–3.97)	0.589
Any Chlamydia trachomatis (CT)^b^	39/249 (15.7%)	7/25 (28.0%)	32/224 (14.3%)	0 (0.0%)	2.32 (0.76–6.43)	0.084

The missing column gives the number of the 249 participants with no recorded value for that variable, and the percentage of 249; denominators in the positive and negative columns vary accordingly.

Effect estimates for categorical variables are odds ratios for TP DNA detection, estimated by conditional maximum likelihood with exact 95% confidence intervals; p-values are from Fisher's exact test, used in preference to the chi-squared test because expected cell counts fell below five in several comparisons.

CI, confidence interval; HIV, Human Immunodeficiency Virus; IQR, interquartile range; SD, standard deviation; STI, sexually transmitted infection; TP, *Treponema pallidum*.

a Complaint of genital, anal or extragenital (oral) lesion.

b Any pathogen detected by PCR in any available sample (oropharyngeal, anorectal or urogenital).

c Hodges-Lehmann estimate of the median difference in age, from the Wilcoxon rank-sum test; a negative value indicates that participants with detected TP DNA were younger.

d Mean difference from Welch's t test, reported as a sensitivity analysis. Age was right-skewed in both groups, so the median and interquartile range are the primary summary, with the mean and standard deviation given alongside.

All 25 participants with detectable TP DNA had an interpretable 23S rRNA sequencing result; resistance-associated A2058G or A2059G mutations were detected in 19/25 (76.0%, 95% CI 54.9–90.6). Resistance-associated mutations were present in 11 of 14 participants with oral-only detection (78.6%, 95% CI 49.2–95.3), 6 of 6 with anal-only detection (100.0%, 95% CI 54.1–100.0) and 2 of 5 with detection at both sites (40.0%, 95% CI 5.3–85.3) (Table 1). These site-specific estimates each rest on fewer than 15 participants and their confidence intervals overlap substantially, so they are presented descriptively and should not be interpreted as evidence of a difference in resistance by anatomical site.

The median VDRL titre was 1:16 (interquartile range 1:8–1:32; range 1:8–1:256). Detection of TP increased with rising VDRL titres, most consistently at oral sites (Fig. 2). For oral-only detection, the proportion rose across every stratum, from 1.1% (95% CI 0.0–6.1) at a titre of 1:8–16.7% (95% CI 2.1–48.4) at 1:128 and 60.0% (95% CI 14.7–94.7) at 1:256, with a similar gradient for any oral or anal detection. This estimate is based on three of five participants in the highest titre stratum and is correspondingly imprecise; it should not be read as a stable estimate of detection at that titre. Tests for trend provided evidence of increasing detection across VDRL titre in all four detection categories (p < 0.001 for oral-only and for any oral or anal detection, p = 0.018 for anal-only, and p = 0.007 for concurrent oral and anal detection; Table 1). For anal-only and concurrent detection, the increase was confined to the lower and middle strata, with no anal-only or concurrent detections among the five participants at 1:256. Each doubling of VDRL titre was associated with an OR for TP DNA detection of 2.44 (95% CI 1.74–3.41) for any oral or anal detection and 2.32 (95% CI 1.56–3.47) for oral-only detection (Table 1). In a sensitivity analysis excluding the 1:256 stratum, evidence for the ordered association persisted for oral-only detection (p = 0.002), any oral or anal detection (p < 0.001), anal-only detection (p = 0.006), and concurrent detection (p = 0.002). Corresponding ORs per doubling of titre were 2.03 (95% CI 1.25–3.28), 2.50 (95% CI 1.70–3.68), 2.29 (95% CI 1.18–4.43), and 2.83 (95% CI 1.30–6.17), respectively.

**Fig. 2:**
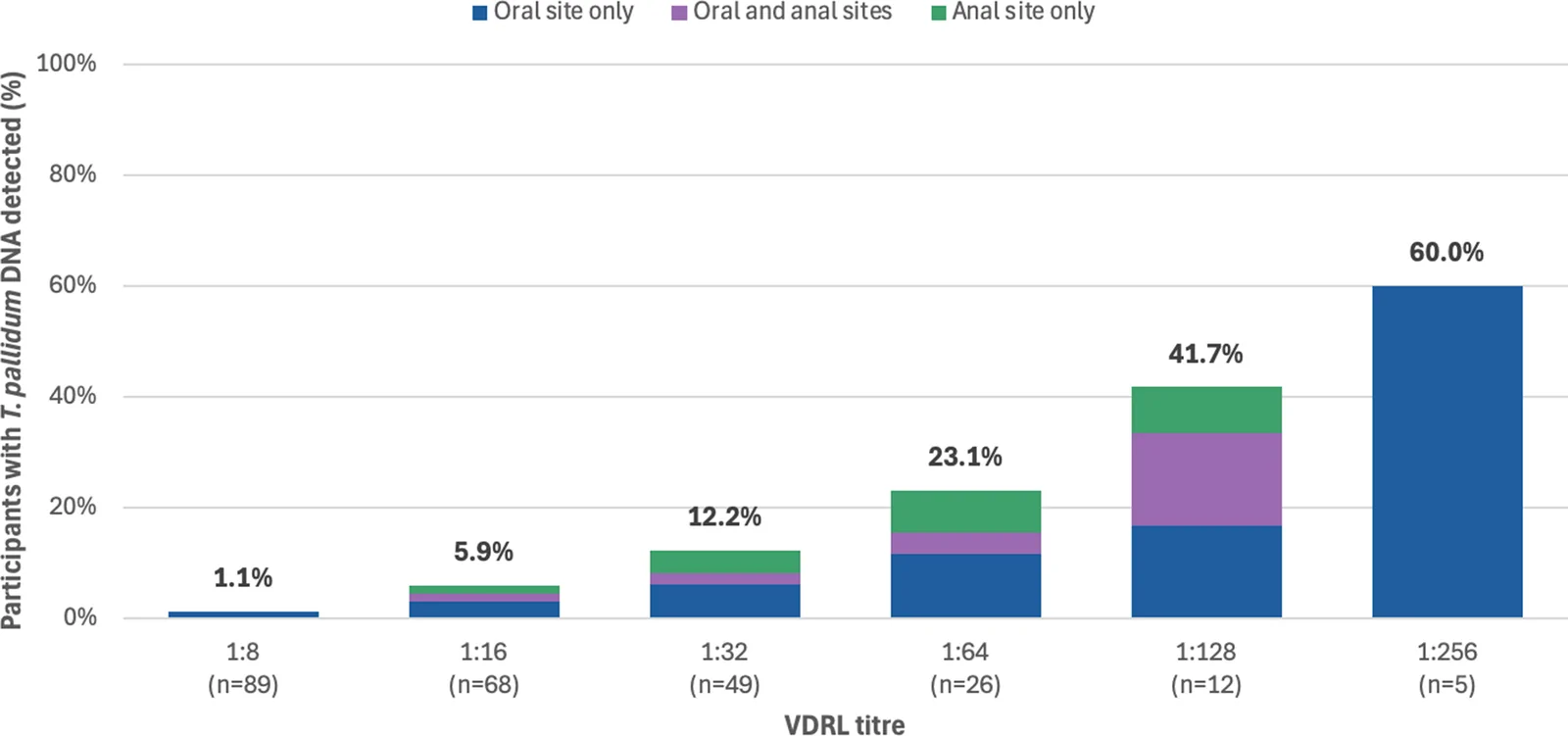
**Detection of *Treponema pallidum* DNA by VDRL titre**. Bars show the proportion of participants with *T. pallidum* DNA detected, by anatomical detection pattern. Detection increased across titre strata (Cochran–Armitage p < 0.001; odds ratio 2.44 [95% CI 1.74–3.41] per doubling of titre). The estimate at a titre of 1:256 is based on three of five participants and is imprecise.

Missing participant characteristic data were uncommon. With the exception of prior syphilis diagnosis, all characteristics had three or fewer missing values (≤1.2%). Prior syphilis diagnosis was missing for 13/249 participants (5.2%), including 3/25 (12.0%) with detectable TP DNA and 10/224 (4.5%) without detection. The corresponding complete-data comparison should therefore be interpreted with particular caution.

## Discussion

The proportion of participants with TP DNA detection (10.0%) is lower than the 25% reported among people with early syphilis.^3^, ^4^, ^6^ Three design features account for most of this difference. Firstly, the populations and recruitment methods differ: earlier studies enrolled cisgender MSM who were attending sexual health services or participating in clinical trials and had been clinically diagnosed with syphilis. In contrast, participants in our study were recruited in the community through respondent-driven sampling and identified by serology alone. Secondly, the clinical stage differs: previous studies were restricted to cases with early syphilis, with the highest detection rates observed in primary and secondary disease cases. However, subgroup analyses within the same studies reported a detection rate of around 10% in patients with early latent infection, a figure quite close to our estimate.^3^, ^4^ Clinical stage was not determined in the present study, however, and oral-specific symptoms were not systematically elicited, so direct comparison with stage-defined populations should be made cautiously. Thirdly, the sampling sites differ: earlier studies tested lesion exudate, blood or urine alongside mucosal swabs. Lesion specimens carry the highest yield; however, our analysis was restricted to two mucosal sites, and the very few lesions were not swabbed. Taken together, these differences suggest that our estimate reflects a population with a low proportion of clinically evident early syphilis, rather than indicating a lower mucosal detection rate specific to TGW. TP DNA was detected in participants with high-titre syphilis, with oral involvement in 76.0% of participants with detectable TP DNA, showing that mucosal TP DNA was detectable in a proportion of participants identified through community-based testing. One comparable Latin American data come from a Peruvian cohort of 264 participants with clinically diagnosed syphilis, who were recruited from public HIV and STI clinics.^7^ Of these participants, 88% had an initial RPR titre of 1:8 or higher. TP DNA was detected in 98 out of 260 participants (37.7%), including 8.5% of 258 oral swabs and 38.8% of 116 anal swabs. This is compared with 10% of participants and 7.9% and 4.6% of oral and anal specimens, respectively, in our study. This difference is consistent with the three design features outlined above: participants were identified clinically rather than through serological screening; lesion swabs were collected alongside mucosal swabs; and anal swabs were only taken from participants enrolled after November 2020. No estimate of mucosal TP DNA detection specific to TGW has been reported, and the Peruvian cohort was not described by gender identity.

Mean Ct values were lower in anal than in oral specimens for both targets. Although lower Ct values may be compatible with a greater quantity of target DNA, the small number of positive specimens, absence of absolute quantification, and potential differences in specimen collection preclude interpreting these values as evidence of a higher treponemal burden at the anal site. The direction of this observation is consistent with previous reports of lower Ct values or higher TP DNA burden at the anus than in the oral cavity.^16^ Whether it reflects differences in sampling efficiency or a genuine site-specific difference cannot be resolved by our design.

A high proportion of participants with detectable TP DNA had TP containing mutations associated with azithromycin resistance. The observed frequency is consistent with reports of high frequencies of these resistance-associated mutations in several regions, including Europe, North America, and China.^14^, ^17^, ^18^ Such mutations have also previously been reported in Brazil,^19^ whereas a recent Mexican study using the same serological definition detected TP DNA in whole blood in 16.7% of asymptomatic participants and found a macrolide resistance mutation in only one of eleven interpretable sequences.^11^ This estimate is based on a small number of sequences and a different specimen compartment, so the comparison with our rate of 76.0% should be interpreted with caution. This observation suggests that assumptions about resistance should be made at a regional rather than a global level and supports continued use of molecular surveillance in Latin America. Current Brazilian Ministry of Health guidelines recommend benzathine benzylpenicillin as first-line treatment for syphilis and do not include azithromycin as a treatment option.^20^ The high frequency of resistance-associated mutations observed in this study is therefore consistent with existing national guidance and highlights the value of continued molecular surveillance of TP in Brazil and Latin America.

TP DNA detection also increased with rising VDRL titres, most clearly for oral and any-site detection. For any oral or anal detection, each doubling of VDRL titre was associated with higher odds of TP DNA detection (OR 2.44, 95% CI 1.74–3.41). Higher non-treponemal titres may reflect potentially greater organism burden, but clinical stage was not determined and PCR detection does not establish dissemination, viability, or infectiousness. This association is nevertheless consistent with previous evidence that TP burden differs across stages and activity of syphilis.^5^ Similarly, in our study, participants with TP detection were younger than those without TP detection, further supporting the hypothesis that host or behavioural factors associated with younger age may be associated with a higher likelihood of mucosal TP DNA detection.^5^

This study focused on TGW, who remain underrepresented in syphilis epidemiology studies despite a substantial burden of STIs. Existing data on mucosal TP DNA detection come primarily from studies conducted among cisgender MSM, limiting their applicability to TGW. Beyond syphilis, systematic three-site compared with vaginal-only sampling for NG and CT in non-sex-worker women attending French STI centres has shown that extragenital-only positivity is not negligible, supporting the broader epidemiological relevance of extragenital sampling in women.^21^ Although these findings concern different pathogens and a different population, they reinforce the importance of considering extragenital anatomical sites in STI research. By specifically examining TGW in Brazil, the present study contributes data on mucosal TP DNA detection in a population that has been underrepresented in previous molecular studies.

This study had several limitations. The small number of participants with detectable TP DNA (n = 25) limits the precision of subgroup analyses, as reflected in the width of the confidence intervals in Table 1, and precluded meaningful multivariable adjustment; the associations reported are therefore unadjusted and may be partly or wholly explained by confounding, including age and correlated behavioural characteristics. The direction and consistency of the titre gradient are better supported than the magnitude of the estimates at its upper end. The cross-sectional design precludes assessment of temporal patterns of mucosal TP DNA detection. PCR detection identifies bacterial DNA but does not confirm organism viability or infectiousness. Specimens had been stored at −20 °C for four to five years prior to TP DNA testing. As prolonged frozen storage can degrade TP DNA, the detection proportions reported here should be regarded as minimum estimates. Rather than relying on a laboratory-based treponemal assay, testing relied on a rapid point-of-care assay, with FTA-Abs only being performed for discordant results. This may have led to some misclassification against the serological case definition. Clinical stage of syphilis could not be reliably determined, owing in part to incomplete clinical examination and the absence of anoscopy or lesion-directed TP testing. Consequently, the high-titre serological definition should not be interpreted as a clinical stage. As neither blood nor lesion specimens were tested by PCR, detection was restricted to two mucosal sites, which makes it difficult to compare this study with those that sampled blood or lesions.^22^ Oral-specific symptoms were also not systematically elicited, limiting our ability to characterise participants as symptomatic or asymptomatic at the oral site. Although missing data were uncommon overall, a history of prior syphilis diagnosis was missing for 13 participants and disproportionately so among those with detectable TP DNA, so the complete-case estimate for that characteristic may be biased. Self-sampling is acceptable and feasible among TGW,^9^ but collection method was chosen by participants rather than randomised and evidence on the comparability of self- and provider-collected oral and anal swabs for TP DNA remains limited; the comparison by collection method should therefore not be read as evidence of equivalence. Finally, although TransOdara used RDS across five Brazilian cities, the present analysis was also restricted to participants with high-titre syphilis and at least one valid stored oral or anal specimen, and used unweighted data from this selected MyTTrA subset despite RDS recruitment in the parent study. The findings should therefore not be interpreted as nationally representative of TGW in Brazil and apply only to TGW without extrapolation to gender-diverse populations more broadly.

In conclusion, TP DNA was detected in oral or anal samples from 10% of TGW with high-titre syphilis, with oral detection more frequent than anal detection. Most participants with detectable TP DNA did not report current STI symptoms, although oral-specific symptoms were not systematically assessed and clinical examination was incomplete. These findings add to the limited evidence on mucosal TP DNA detection among TGW and support the importance of epidemiological and molecular surveillance in populations with a high burden of syphilis. The high frequency of azithromycin resistance highlights the value of continued molecular surveillance and is consistent with current Brazilian guidelines, which recommend benzathine benzylpenicillin as first-line treatment and do not recommend azithromycin for syphilis.

## Contributors

RS: Data interpretation, original draft writing—review and editing.

DJM: Conceptualisation, methodology, data organisation, statistical analysis support, writing—review and editing.

PAG: Laboratory methodology, supervision, writing–review and editing.

PMe: Conceptualisation, statistical analysis—review and editing.

SC: review and editing.

LM: TransOdara study team—review and editing.

BB: Conceptualisation, methodology, supervision–review and editing.

MAV: Conceptualisation, methodology, supervision, funding acquisition, writing—review and editing.

CB: Conceptualisation, methodology, supervision, funding acquisition, writing—review and editing.

PG: review and editing.

PMa: Conceptualisation, methodology, supervision, funding acquisition, writing—review and editing.

ND: Conceptualisation, methodology, supervision, funding acquisition, writing—review and editing; All authors read and approved the final manuscript.

DJM and PMe accessed and verified the underlying data. All authors had full access to the data reported in the study, read and approved the final manuscript, and accept responsibility for the decision to submit for publication. The MyTTrA Study group contributed to study design, specimen collection and laboratory analysis, and reviewed the manuscript.

## Data sharing statement

De-identified quantitative data may be available from the MyTTrA Study Steering Group on reasonable request via the corresponding authors.

## Declaration of interests

DJM reports consulting fees from the World Health Organization (Department of HIV, TB, viral hepatitis and STIs) and an unpaid board membership of Gay Health Network, Ireland, a registered Irish charity. MAV reports a research grant from the National Council for Scientific and Technological Development (CNPq), honoraria from Gilead Sciences for an educational event at AIDS 2026, an unpaid coordination role at NUDHES, and unpaid membership of the International Advisory Committee of the Millennium Nucleus DIBASS. All other authors declare no competing interests.
